# Exploring physician specialist response rates to web-based surveys

**DOI:** 10.1186/s12874-015-0016-z

**Published:** 2015-04-09

**Authors:** Ceara Tess Cunningham, Hude Quan, Brenda Hemmelgarn, Tom Noseworthy, Cynthia A Beck, Elijah Dixon, Susan Samuel, William A Ghali, Lindsay L Sykes, Nathalie Jetté

**Affiliations:** Department of Community Health Sciences, University of Calgary, Calgary, AB Canada; Department of Medicine, University of Calgary, Calgary, AB Canada; Department of Psychiatry, University of Calgary, Calgary, AB Canada; Department of Pediatric Nephrology, University of Calgary, Calgary, AB Canada; Department of Clinical Neurosciences, Hotchkiss Brain Institute and Institute for Public Health, University of Calgary, Calgary, AB Canada

**Keywords:** Survey methodologies, Healthcare, Response rate, Specialists, Physicians

## Abstract

**Background:**

Survey research in healthcare is an important tool to collect information about healthcare delivery, service use and overall issues relating to quality of care. Unfortunately, physicians are often a group with low survey response rates and little research has looked at response rates among physician specialists. For these reasons, the purpose of this project was to explore survey response rates among physician specialists in a large metropolitan Canadian city.

**Methods:**

As part of a larger project to look at physician payment plans, an online survey about medical billing practices was distributed to 904 physicians from various medical specialties. The primary method for physicians to complete the survey was via the Internet using a well-known and established survey company (www.surveymonkey.com). Multiple methods were used to encourage survey response such as individual personalized email invitations, multiple reminders, and a draw for three gift certificate prizes were used to increase response rate. Descriptive statistics were used to assess response rates and reasons for non-response.

**Results:**

Overall survey response rate was 35.0%. Response rates varied by specialty: Neurology/neurosurgery (46.6%); internal medicine (42.9%); general surgery (29.6%); pediatrics (29.2%); and psychiatry (27.1%). Non-respondents listed lack of time/survey burden as the main reason for not responding to our survey.

**Conclusions:**

Our survey results provide a look into the challenges of collecting healthcare research where response rates to surveys are often low. The findings presented here should help researchers in planning future survey based studies. Findings from this study and others suggest smaller monetary incentives for each individual may be a more appropriate way to increase response rates.

## Background

With the rise of the Internet and email in recent decades, online and web-based tools offer promising advances for healthcare survey research methods [1,2]. Immediate survey delivery, real-time data tracking and inexpensive costs are selling points of email or web-based surveys [2]. Electronic surveys may also increase response rates through ease of access, as well as greater individual anonymity compared to face-to-face or telephone interviews [2,3]. Despite the increased use of web-based surveys, considerable debate about the success and usefulness of this type of survey mode exists [4-6]. Studies using both email and mail paper surveys demonstrate conflicting evidence as to whether email surpasses mail as a delivery modality [6]. Survey response rates have also in general been on the decline for the past decade in the field of health related research [1,6-8].

Despite the declining use of survey research methods, this type of research remains an important way of research to gather information about physicians’ knowledge, attitudes, and to evaluate the impact of clinical research on practice [9]. Soliciting physician input is also essential when existing healthcare policies are being updated or to inform new policies [10]. Unfortunately, physicians are a professional group with low survey response rates in general [11,12]. While family doctors have typically had low survey response rates [12,13], specialist physicians historically have demonstrated variability in response rates [14-17]. Kellerman and Herold [8] reviewed the variability of demographic characteristics on physician responses to surveys and found that medical specialty type was not associated with response rates. There is a clear dispute in the literature as to whether survey recruitment methods that are successful with general practitioners are also successful with other physician specialties. Also it is actually uncommon in the survey methodology literature to see surveys conducted across multiple physician specialties. Our study allowed us to look at variations in response rate across multiple specialties and in addition, our recruitment methodology was unique in that individualized/personalized emails were sent to each physician. As survey research with specialist physician groups is on the rise [18-20], more survey research involving physician specialists is needed in order to understand the reasons for differences in response rates within this medical group. Specifically, identifying new recruitment methods that may be uniquely related to the physician specialist groups is needed.

Differential effects by medical specialty may be the result of several factors including preference for survey mode, survey design, survey length and potential confounding factors such as the gender of respondents [6,8,21]. For example, in one survey study of family and specialist physicians, pediatricians not only had higher response rates overall and within the promised-incentive group but were also the least sensitive to the timing of the incentive. One possible reason for this is that the response rate may be confounded by gender; women may be more likely to respond to surveys than men and women make up a larger proportion of pediatricians [21].

Response rate can be further affected by the survey topics. When the topic is of high interest to respondents, potential respondents are more likely to respond to the survey [6,22-24]. In addition, whether survey topics are sensitive or non-sensitive or concern attitude or fact is likely to affect response rates in web surveys [6,22,24]. For example, obtaining data on physicians’ billing practices is often challenging due to the sensitive nature of the topic [25]. Only a handful of studies have examined and compared survey response rates among physician specialists, in whom different survey methodologies may be necessary to achieve acceptable response rate [14,26].

As part of a larger project assessing the impact of physician billing practices on the completeness of administrative data, we sought to examine response rates to a web-based survey developed for medical and surgical specialists. The survey was designed to gather demographic and billing information from physician specialists in Fee-for-Service (FFS) and Alternative Payment Plans (APP). The recent introduction of Alternative Payment Plans across Canada has changed the way that many physicians are reimbursed and subsequently the process of physician billing [27]. Physicians on APPs are encouraged to submit claims for the services they provide called “shadow bills”, for administrative purposes. Unfortunately, with these new APPs, they are not compensated for the time spent recording the services they deliver (i.e. shadow billing).

The objective of our study was to assess billing practices amongst a large group of medical and surgical specialists using a modified version of the Dillman method [23,28]. Our hypothesis was that the response rate to our web-based survey would be low due to the sensitive nature of the topic under investigation (i.e. physician billing practices). Here we discuss the results from our study findings including survey methodology and response rates, and we explore reasons for non-response.

## Methods

### Physicians’ survey

Survey design: The initial three-page survey was reviewed and refined for content validity by a working group of eight senior researchers and practicing physicians from the various relevant specialties [i.e. intensive care unit (ICU), internal medicine, neurology and neurosurgery, pediatrics, psychiatry and general surgery]. The refinement process included a content review from all stakeholders listed as co-investigators for the project.

The final survey gathered information regarding physicians’ billing status (FFS versus APP), whether they are obligated to shadow bill as part of their APP contract (if applicable), whether incentives are provided to them to shadow bill (if applicable) and demographic information. The email survey design and layout included two pages of questions. The primary method of accessing and completing the survey was via the Internet using a well-known and established survey company (www.surveymonkey.com). The survey company hosted and collected the survey data and only participants who were sent the email could connect to the hyperlink and respond to the questionnaire. However, if physicians had trouble accessing or completing the online survey, a paper version could be requested by mail or fax. Ethical approval for the study was obtained from the Office of Medical Bioethics at the University where the study took place out of.

Survey participants: The following physician specialties were targeted: ICU, internal medicine, neurology and neurosurgery, pediatrics, psychiatry and general surgery. We restricted our sample to these specialties as they were established APP and FFS programs and their specialty is a potential confounding factor for shadow billing behaviour and these specialty groups had a large number of registered physicians in a large Canadian city where the study took place.

Inclusion criteria were: 1) physicians employed and practicing in 2009; 2) on an APP or FFS payment plan and; 3) physicians providing inpatient or outpatient (i.e. clinic) services based at one of the four acute care hospitals in the city where the study took place. Exclusion criteria were: 1) general practitioners (as the majority are remunerated by FFS system and did not fit into the scope or budget of our study) and; 2) medical trainees (i.e. medical students, residents, and fellows) as the majority of them do not submit billings.

Respondent Sampling: The survey sampling frame was generated using a list of physicians from the 2008 Canadian Medical Directory. The original list included 1012 physicians, their clinic/hospital appointment, specialty, as well as contact information. Because the contact information on the list is not updated regularly, the information (i.e. phone, address, email) and specialty was further verified through the latest faculty/department contact lists, and physician contact directories posted on websites of Alberta Health Services, hospitals and the College of Physicians of Alberta website. After the verification of contact information, 108 physicians were excluded due to incorrect contact information or unavailable contact information. The final population of physicians targeted included 904 physicians (324 internists, 58 neurologists/neurosurgeons, 171 pediatricians, 118 psychiatrists and 233 general surgeons).

### Survey administration and recruitment strategy

**Figure 1:**
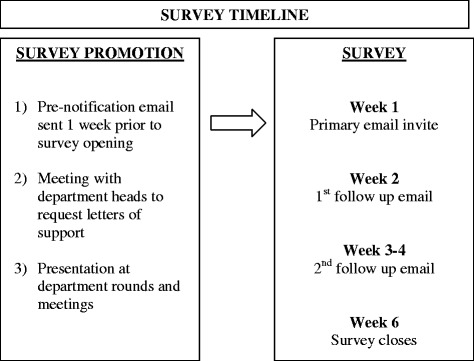
**Methodology of survey invitation.**

Survey promotion and process: Figure 1 outlines the survey invitation process and timeline. A website containing the project information, investigators’ contact information, and a link to the survey was developed. Meetings were held with department heads for each medical/surgical group to discuss the study and obtain letters of support. Meetings and presentations promoting the survey were also organized with the various medical departments. All the initial email invitations contained a link to the study website which provided further information regarding the study and research team, and a link to access the survey. Additionally, all emails contained the eligibility criteria for participating physicians, the opening and closing dates for access to the survey, and a unique identification number for each participant.

The main strategy to promote the website and survey was to involve key individuals (i.e. influential physicians from each medical group included in the study) to facilitate, encourage, and support their colleagues, department heads, and other physicians to complete the survey. The emails were addressed and sent individually to each physician by name, thereby avoiding any issues with confidentiality which can be a challenge with mass emailing lists. Emails were also sent using personalized subject headings. The key physicians who were supporting the promotion of the survey authorized their names to be used in the subject heading (i.e. Dr. X is asking for your help). Supporting physicians’ names used in the subject heading were representative of all the medical departments/specialties involved in the study.

The goal of using a familiar physician’s name to personalize the invitation was to add peer influence and to increase the likelihood of physicians’ reading the email and consenting to participate. A generic subject heading listing the funding body (i.e. xxx funded research project) was also used in cases where the use of an individual physician’s name was not deemed appropriate. All responding inquiries or comments were directed primarily to the lead research coordinator and physician co-investigators. Physicians were given the opportunity to submit their unique study ID number with the submission of their survey, and by doing so their names were entered into a one-time draw for three $200.00 (Canadian) gift certificates to a local bookstore.

Finally, after the allotted time period passed to respond to the initial survey invite, a secondary survey was sent to all eligible non-respondents asking them to identify the reasons for not participating in the original survey. The actual question that was sent via email was “In a few words or sentences, please indicate why you were unable or unwilling to complete the survey.” Physician specialist responses were gathered qualitatively.

### Statistical analysis

Descriptive statistics were used to describe specialty response rates and reasons for non-response in current case study. Chi-square analysis was used to examine differences between specialists by demographic characteristics or payment plan group. We compiled results from a secondary survey examining physicians’ reasons for non-response and categorized comments into five broader themes based on degrees of commonality within responses. All statistical analyses were conducted using Stata IC, Version 12 (StataCorp LP, College Station, TX).

## Results

### Initial physician survey

Of the 904 eligible physicians contacted, 317 eligible physicians responded to the survey, for an overall response rate of 35.0%. Table 1 outlines the baseline demographic characteristics of the survey respondents. The majority of respondents were male (55.1%) between the ages of 40 and 59 (51%). A large proportion of physicians had been in practice for more than 15 years (44.6%), with only 18.3% in practice less than 5 years. The majority of physicians (47.7%) were remunerated on a full-time APP plan and 38.1% were paid on a FFS plan (see Table 1).

**Table 1:** **Demographic characteristic of survey respondents**

**Physician specialty**	**Physicians (% of 312)**
Surgery	28.2
Internal medicine	44.6
Paediatrics	18.0
Psychiatry	9.3
Age*	
30-39 years	22.1
40-59 years	51.0
More than 59 years	9.6
Missing data	17.3
Sex*	
Male	55.1
Female	27.6
Missing data	17.3
**Length of practice**	
<5 year	18.3
5-14 years	37.2
≥15 years	44.6
**Payment plan**	
Fee-for-Service	38.1
Alternative Payment Plan (Part)*	14.1
Alternative Payment Plan (Full)*	47.7

*Note: Alternative Payment Program (APP), Fee-For-Service Payment Program (FFS).

*Age percentage calculated among individuals with non-missing data; 54 individuals (17.3% of total sample) did not provide information.

*Sex percentage calculated among individuals with non-missing data; 54 individuals (17.3% of total sample) did not provide information.

Table 2 outlines the characteristics of physician survey respondents by type of payment plan. Internal medicine specialists on a full-time APP plan were most likely to respond (54%), followed by internal medicine specialists on a part-time APP plan (38.6%) and surgery (35.3%) and internal medicine (35.3%) specialists on a FFS plan. Psychiatrists on both full-time APP (8.7%) and FFS (9.2%) plans were least likely to respond to the survey. Physician specialists aged 40–59 years on both APP part-time (63.3%) and full-time (51%) plans were more likely to respond to the survey. FFS physicians (46.2%) in the 40–59 years of age category were also more likely to respond than other age categories. Male physician specialists had statistically significant higher levels of response across all payment plans (APP full 51.7%; APP part 54.5%; FFS 59.7%) compared to their female counterparts. Finally, those physician specialists with more than 15 years in practicing medicine were more likely to respond. Part-time APP physicians (50%) with the highest response rate followed by FFS (47%) and full-time APP physicians (41%).

**Table 2:** **Characteristic of physician specialists by payment program**

	*FFS (% of 119)	*Part APP (% of 49)	*Full APP (% of 149)	**P-value**
**Proportion of physicians**	38.1	14.1	47.7	
**Physician specialty**				
Surgery	35.3	27.3	22.8	
Internal medicine	35.3	38.6	54.0	
Paediatrics	20.2	22.7	14.8	
Psychiatry	9.2	11.4	8.7	0.217
Age*				
30-39 years	22.7	15.9	23.5	0.174
40-59 years	46.2	63.3	51.0	
More than 59 years	9.2	2.2	12.1	
Missing data	21.8	18.2	13.4	
Sex*				
Male	59.7	54.5	51.7	<0.05
Female	18.5	27.3	34.9	
Missing data	21.8	18.2	13.4	
**Length of practice**				
<5 year	37.0	38.6	36.9	0.456
5-14 years	16.0	11.4	22.1	
≥15 years	47.0	50.0	41.0	

*Note: Alternative Payment Program (APP), Fee-For-Service Payment Program (FFS).

*Age percentage calculated among individuals with non-missing data; 54 individuals (17.3% of total sample) did not provide information.

*Sex percentage calculated among individuals with non-missing data; 54 individuals (17.3% of total sample) did not provide information.

The response rates by timing of reminders for the medical specialty groups are shown in Table 3. Internal medicine (33.6%) and neurology/neurosurgery (34.5%) had the highest response rates following the 1^st^ follow-up/reminder email. Following the 2^nd^ follow-up/reminder email, general surgery and pediatrics response rates increased the most. Overall response rates obtained by specialties were: Neurology/neurosurgery (46.6%), internal medicine (42.9%), general surgery (29.6%), pediatrics (29.2%) and psychiatry (27.1%).

**Table 3:** **Shadow billing survey response rates per medical specialty**

	**Internal medicine total** ***n*** **= 324 N (%)**	**Neurology/neurosurgery total** ***n*** **= 58 N (%)**	**Pediatrics total** ***n*** **= 171 N (%)**	**Psychiatry total** ***n*** **= 118 N (%)**	**General surgery total** ***n*** **= 233 N (%)**
*Response rate after 1^st^ email follow up (1 week)	109 (33.6%)	20 (34.5%)	12 (7.0%)	9 (7.6%)	31 (13.3%)
Response rate after 2^nd^ follow up (3–4 week)	134 (41.4%)	25 (43.1%)	50 (29.2%)	22 (18.6%)	66 (28.3%)
Total response rate(%)	139 (42.9%)	27 (46.6%)	50 (29.2%)	32 (27.1%)	69 (29.6)

*Overall response rate was 35.0% (317/904), total response rate (%) row is a result of additive responses from first and second email follow up reminders.

Of the physicians who responded to the initial survey, 82.6% (262/317) provided their unique identification number to be entered into the lottery draw.

### Follow up survey of non-respondents

Sixty-three physicians responded to the secondary survey aimed at exploring reasons for non-response, for a response rate of 11.8% (n = 63/533). Of those who responded to the follow-up survey (n = 63), 70.5% were males and 29.5% were females. Respondents for this follow-up survey were from the following specialties: Internal medicine (34.2%), general surgery (27.8%), neurology/neurosurgery (0.03%), pediatrics (18.2%) and psychiatry (16.9%). Reasons for non-response were survey burden, with 60.3% of respondents reporting that there were too many survey requests and they lacked the time to complete them; 15.9% believed they were not eligible; 12.7% had no interest or saw no benefit to completing the survey; 7.9% felt the survey was asking information which was too private; and, 3.2% did not know their billing mechanism in order to complete the survey. It should be noted that in the current study, a handful of physicians (n = 5) responded unfavorably to the offer of a lottery draw incentive, finding it offensive and unethical.

## Discussion

We conducted an online survey using a personalized invitation email strategy (with web-based survey) in addition to various other recruitment methods (multiple follow-up/reminders, lottery draw). These strategies have been used in prior survey studies to increase physician response rates [29,30]. The 35.0% response rate for our own survey was lower than anticipated, but in view of the sensitive nature of the topic under investigation, it was not unexpected. Our response rate is still higher or comparable to similar studies using email as a distribution mode among physician specialists [31,32].

The sensitive nature of our topic (i.e. physician billing practices) and the time-period during which our survey was conducted most likely contributed to a lower response rate. Survey research shows that survey topics which are sensitive or non-sensitive or concern attitude or fact is likely to affect response rates in web surveys [6,24]. According to several meta-analyses, the salience of a topic is one of the most important factors that influence response rates in both mail and web surveys [6,22,23,33,34]. The contract renewal period for all physicians’ on APPs occurred during the months in which our survey opened. This may have affected response rates, as physicians may have been cautious about consenting to have their billing practices reviewed for the purposes of our study, as the contract renewal process directly examined the quality of physician’s billing submissions. Despite this challenge, many of the responding physicians contacted us to ask questions about our study after the initial survey invitation, indicating they were interested in our study topic and requested having the results sent to them. In one case, a face-to-face interview was set up with a physician to discuss the details of the study. Given our project resources, email was the most efficient, inexpensive and timely manner of contacting survey participants. Tracking, managing, and organizing the incoming data was simple and was ideal for the short project timeframe.

Results from studies of previous web-based surveys indicate similar trends in response rates to the current study. In one meta-analyses, the mean response rate for 68 web-based surveys reported in 49 studies was 39.6% [22], similar to our current findings. In Kellerman et al. [8], the response rates for general practitioners and specialists were 40.1% (186/464) and 49.6% (235/474), respectively. Response rates among specialist physicians vary within the literature [21]. In one study, a mail survey, pediatricians had higher response rates compared to general practitioners, internists and obstetrics-gynecology physicians and were also the least sensitive to the timing of the incentive [21]. In the current study, neurologists and internists had the highest levels of response. This may also be due to gender differences in that a large proportion of women in those specialties responded to the questionnaire compared to the other specialties. Our findings are in concordance with previous literature which suggests women physicians may be more likely to respond to surveys than male physicians [6,21]. Additionally in the current study, internal medicine and pediatrics had some of the most longstanding Alternative Payment Programs (i.e. established in 2003–2005), which may have lessened the concern of physicians in responding to a survey about billing behaviors making them more likely to respond compared to the other specialties.

Among published studies involving physician as respondents, survey response rates seem to be most influenced by the use of individual monetary incentives [35-37]. For example, a US study found that the provision of a small ($2.00) monetary incentive sent to each physician invited to participate, yielded a substantially greater response rate (56.0% vs. 44.0 %) than the lottery draw of a larger, one-time, cash incentive [38]. It has been proposed that in order to achieve the response rates needed to validate health care policy-related research using survey methodology, the offer of monetary incentives may become a necessary part of the research process [39,40]. A recent Canadian study examining the use of a substantial monetary lottery incentive among physicians did not find that financial incentives improved response rate. In fact, response rates were lower in the following year (35.9% in 2004, 31.6% in 2007) [41]. In a study examining prepaid incentives to physician specialists, the response rate was 52.1% for physicians who received a $20 check versus 67.8% for physicians who received a $50 check (P < 0.001) [42]. As physicians become increasingly burdened with surveys, studies suggest larger incentives may be necessary to engage potential respondents and thus maximize response rate [42].

Based on these findings and from our own survey findings, individual smaller financial incentives for each respondent may increase initial buy-in from participants, and may be superior to large, one-time lottery draws. However since we did not include a comparison group of individual small financial incentives, this conclusion is somewhat limited. Additionally it is important to note, that a handful of physicians (n = 5) responded to the offer of a lottery draw incentive unfavorably. Similar studies have found negative responses to incentives [26,43], although not the point of withdrawing from the study. In the case of the current study, the physicians declined to participate as a result of being offered a personal incentive.

The timing of follow up reminders has also been shown to increase response rates. However, recommendations regarding the timing of follow up and frequency of follow up reminders vary substantially in the literature [13]. Our study was associated with an increase in response rates by medical or surgical specialty after each follow-up/reminder; however, no clear pattern surfaced as to which timeframe (1^st^ week, 3^rd^ week) is most ideal to increase response rates. Our results suggest that at least one follow-up reminder may prove beneficial in increasing response rates [4,44]. More recently, research suggests too many reminders may be viewed as possible harassment of potential respondents [45]. Future research should focus on the ideal number and nature of reminders and specifically, how much is too much.

Researchers must always explore and address the bias associated with non-response. Physicians who responded to our follow up survey (11.8%) displayed similar patterns or characteristics of response across gender and specialty. However, beyond these two characteristics, we were not able to compare or contrast other factors that may have influenced certain physicians to respond versus non-respondents. Kellerman and Herold [8] outline the reasons why responding and non-responding physicians tend to share similar characteristics. Physicians as a group are more homogeneous regarding knowledge, training, attitudes, and behavior and variations that do exist among physicians may not be as associated with willingness to respond or survey content [8].

In the current study, it is important to recognize that non-respondents may differ from participating physicians in ways we were unable to assess and is noted as a possible response bias issue. The main reason for initial non-response in our study was survey burden, with a lack of time to complete them (60.3%). Physicians commonly acknowledge that too many survey requests and growing constraints on their time limit their ability to participate in multiple, concurrent survey- based studies [46] . Given the demands on their time, survey topic or salience must be relevant and the survey must present a benefit to physicians in order for them to participate. Studies show physicians are interested in endorsing certain aspects of research where the opportunity to enact quality improvement and contribute to clinical knowledge is evident [47].

It was hypothesized that using personalized email subject headings with the names of key physicians who were supporting the promotion of the survey (i.e. Dr. X is asking for your help) would help bolster participation. As with mail surveys, previous literature indicates using personalized correspondence is apparently associated with higher response rates for electronic survey [22]. However this also may have led to response bias in the likelihood of increased participation of physicians in certain specialties (i.e. if the respondent was familiar with the physician who was promoting study). However, a generic subject heading listing the funding body (i.e. xxx funded research project) was also used in cases where the use of an individual physician’s name was not deemed appropriate. The authors feel this bias would not have affected the results in a significant fashion, especially given the low response rate.

It is important to discuss the limitations of our study. First, 108 physicians were excluded due to incorrect contact information or unavailable contact information, resulting in possible selection bias. Second, there is always the possibility that an email will be identified as “spam mail” when using email as a contact method, possibly further reducing the response rate. Third, we only used one survey mode (i.e. email) which may have limited our response rate. Other limitations included the lack of a comparison group to establish whether our personal survey method enhanced response rates. As there were no controlled groups to compare various interventions that may be associated with improved response rate, it is not possible to firmly establish definite drivers of the degree of response observed in this study. However, we attempted to explore the different reasons for non-responses among physicians in our survey. Finally, the survey was limited to one large metropolitan city in Canada; thus, the findings may not be generalizable to other geographical locations, or to general practitioners or physicians in training.

## Conclusions

In conclusion, our online survey response rate of 35.0% remains comparable to response rates from previously published physician specialist survey-based studies. Variations across medical specialties may have been influenced by gender as woman in certain specialties (i.e. internal medicine and neurology) were more likely to respond than their male counterparts. The response rate in the current study was likely influenced by the sensitive survey topic, but it is likely that specialties (i.e. pediatrics and internal medicine) with longstanding APP programs were more likely to respond as they had more experience with billing within that program. Future survey studies are needed to determine the ideal methodology based on survey topics for physician specialists. This study shares some of the challenges and successes of conducting survey research among multiple physician specialties, where advancement in successful survey recruitment methods is necessary.

## Abbreviations

APP: Alternative Payment Plans
FFS: Fee-for-Service
ICU: Intensive Care Unit
